# Immunometabolic Interactions in Obesity: Implications for Therapeutic Strategies

**DOI:** 10.3390/biomedicines13061429

**Published:** 2025-06-10

**Authors:** Qin Fei, Jueru Huang, Yi He, Yufeng Zhang, Xiaojun Zhang, Jing Wang, Qiang Fu

**Affiliations:** 1 The Third People’s Hospital of Chengdu, Chengdu 610031, China; feiqin111@outlook.com (Q.F.); hjr6222683@outlook.com (J.H.); hycdssyymzk@outlook.com (Y.H.); zhangyf720@outlook.com (Y.Z.); w6244339@163.com (J.W.); 2 Department of Clinical Laboratory, Honghui Hospital, Xi’an Jiaotong University, Xi’an 710054, China; xiaojunzhangtg@163.com

**Keywords:** obesity, chronic inflammation, immune dysregulation, adipose tissue macrophages, gut microbiota, metabolic disorders, immunomodulation

## Abstract

Obesity is characterized by excessive fat accumulation that triggers chronic low-grade inflammation and systemic immune dysregulation, significantly increasing the risk of metabolic disorders including insulin resistance, type 2 diabetes, and cardiovascular disease. This review examines the bidirectional relationship between obesity and immune dysfunction, focusing on how immune cell infiltration in adipose tissue drives inflammatory processes. We highlight the phenotypic shifts in key immune populations—macrophages polarized toward proinflammatory M1 phenotypes, T cell exhaustion occurrs, and alterations appear in B cells, natural killer (NK) cells, and dendritic cells—that collectively contribute to metabolic deterioration. The gut microbiome emerged as a critical mediator in this relationship, influencing both immune responses and metabolic regulation through gut–liver and gut–brain axes. We explore emerging immunomodulatory therapeutic strategies, including anti-inflammatory agents, microbiota interventions, and targeted immune therapies such as innovative nanomedicine approaches. The review also addresses the challenges of immunotherapy in obesity, particularly the paradoxical effects observed in cancer immunotherapy outcomes and the need for personalized treatment approaches. Artificial intelligence is highlighted as a potential tool to enhance patient stratification and treatment optimization in future immunomodulatory interventions. Understanding these immunometabolic interactions provides a foundation for developing more effective therapeutic strategies that could transform obesity management and reduce the burden of obesity-related metabolic diseases.

## 1. Introduction

The Global Burden of Disease Obesity Collaborators has reported that over 603.7 million adults are obese [1]. Obesity has emerged as a critical public health challenge globally, characterized by an excessive accumulation of body fat that poses significant risks for various metabolic disorders, including type 2 diabetes mellitus (T2DM), cardiovascular diseases (CVD), and non-alcoholic fatty liver disease (NAFLD) [2,3]. The pathophysiology of obesity is complex and multifactorial, involving genetic, environmental, and behavioral factors [4]. However, an increasingly recognized component of obesity is the role of the immune system, particularly the diverse array of immune cells that infiltrate adipose tissue and modulate metabolic processes [5].

Recent studies have elucidated the dual role of immune cells in the context of obesity. On one hand, certain immune cells, such as macrophages, T cells, and B cells, can adopt proinflammatory phenotypes that exacerbate insulin resistance and promote the development of obesity-related complications [5,6]. For instance, the accumulation of M1-like macrophages in adipose tissue is associated with increased secretion of proinflammatory cytokines, which contribute to systemic inflammation and metabolic dysregulation [7,8]. Conversely, other immune cells, including regulatory T cells (Tregs) and alternatively activated macrophages (M2), exhibit anti-inflammatory properties that can mitigate the adverse effects of obesity and promote metabolic health [9,10].

The interplay between these proinflammatory and anti-inflammatory immune cells is critical in determining the overall inflammatory milieu of adipose tissue and its systemic consequences. This dynamic balance suggests that targeted modulation of specific immune cell populations may offer a novel therapeutic strategy for addressing obesity and its associated comorbidities. By selectively enhancing the activity of anti-inflammatory immune cells or inhibiting the proinflammatory pathways, it may be possible to improve metabolic outcomes and provide symptomatic relief from diabetes, cardiovascular disease, and liver disease.

Moreover, the implications of immune cell involvement in obesity extend beyond metabolic disorders to other non-metabolic diseases, including cancer. Chronic inflammation, driven by dysregulated immune responses, has been implicated in tumorigenesis and cancer progression. Therefore, understanding the intricate relationship between immune cells and obesity not only sheds light on potential therapeutic interventions for metabolic diseases but also opens avenues for exploring their role in cancer prevention and treatment.

Based on these emerging insights, this review aims to reinterpret obesity from the perspective of immune dysfunction and chronic inflammation, thereby providing a more comprehensive understanding of the pathophysiological mechanisms of obesity. We systematically identify the key immune cells involved in obesity, highlighting their dual roles in disease progression and metabolic regulation. Furthermore, we explore novel therapeutic strategies targeting immune mechanisms, emphasizing their potential to revolutionize obesity treatment. By integrating these perspectives, this review not only advances our fundamental knowledge of obesity but also lays the foundation for innovative, immune-based interventions to mitigate its health consequences.

## 2. The Interaction Between Obesity and the Immune System

### 2.1. The Effects of Obesity on Immune Disregulation

The interaction between obesity and the immune system has garnered significant attention, particularly regarding its impact on various health outcomes, including increased susceptibility to infections, chronic inflammation, and abnormal immune responses in pathological states [11,12,13]. Research has indicated that obesity triggers a series of immune dysregulations characterised by chronic low-grade inflammation, which fundamentally alter the interactions between immune cells [14,15].

First, obesity has been associated with a proinflammatory state driven by multiple factors, including abnormal adipokine secretion and elevated levels of inflammatory cytokines [16,17]. Studies have confirmed that elevated levels of leptin and C-reactive protein (CRP) enhance inflammatory signalling pathways and amplify immune responses [18,19]. Specifically, the interaction between leptin and immune function plays a key role in mediating inflammatory responses; leptin is not only a key regulator of metabolism but also acts as a proinflammatory cytokine to activate immune cells [20,21]. This disruption involves both the innate and adaptive immune systems. Studies have shown that impaired immune function, such as reduced chemotaxis and abnormal macrophage differentiation, leads to an immunosuppressed state, which is common in obese individuals [22,23,24].

Furthermore, specific mechanisms by which obesity influences immune responses have been identified. For example, adipose tissue macrophages (ATMs) are important immune cells significantly affected during obesity, and their polarization changes enhance inflammatory responses. These ATMs promote systemic inflammation by increasing the production of cytokines such as tumor necrosis factor-alpha (TNF-α) and interleukin 6 (IL-6), thereby maintaining a vicious cycle of immune dysfunction [25]. Additionally, evidence suggests that obesity impairs the generation and function of antigen-specific T cells, which are crucial for effective immune memory, thereby weakening the host’s ability to mount an effective immune response to infections [26]. This phenomenon is particularly pronounced in the context of viral infections, where obese individuals exhibit exacerbated immune responses leading to adverse outcomes, as was observed during the Coronavirus Disease 2019 (COVID-19) pandemic [27]. Additionally, studies have shown that adipocytes secrete proinflammatory cytokines that disrupt immune homeostasis, resulting in impaired T cell function and weakened B cell-mediated antibody responses [28,29]. Furthermore, dendritic cells, which play a crucial role in initiating and regulating immune responses, exhibit dysfunction in obese individuals. These cells increase in number within adipose tissue and exhibit enhanced activation states, leading to impaired differentiation of helper T cells and dysfunction of CD4+ T cells [30]. Furthermore, immune responses in obesity are exacerbated by abnormal cytokine secretion from adipose tissue stromal cells (e.g., interleukin 33 (IL-33)), which impairs the development and functional capacity of Tregs and B cells in an inflammatory environment [31,32]. These findings collectively indicate that obesity could profoundly reshape immune responses through mechanisms involving multiple immune cells, highlighting the importance of understanding these interactions for developing clinical strategies to improve obesity-related diseases and immunotherapies.

Additionally, emerging research has highlighted the impact of obesity on complex interactions within the immune ecosystem. For example, changes in the infiltration and functional adaptability of immune cells (such as natural killer cells (NK cells) and T cells) suggest that obesity increases susceptibility to infection by disrupting their training and activation processes [33,34]. Macrophage polarization, particularly towards the M1 inflammatory phenotype, is directly associated with enhanced inflammatory responses, revealing that obesity exacerbates the pathological processes of chronic diseases such as diabetes and cardiovascular disease through distinct mechanisms [35]. To better illustrate the distinct roles of these immune cells in obesity-related inflammation, Table 1 summarizes their key functions, alterations in the obesity status, and the resulting impact on metabolic and immune homeostasis.

Overall, the relationship between obesity and immunity is determined by complex interactions that promote chronic inflammation and altered immune function, potentially leading to increased susceptibility to infection and chronic diseases. Understanding these mechanisms is crucial for developing targeted therapeutic strategies to mitigate the adverse health outcomes associated with obesity. 

Recent studies have further revealed the fine-grained mechanisms of metabolic reprogramming of immune cells in the obese state [43,44,45]. Single-cell RNA sequencing and spatial transcriptomics analyses reveal that different immune cell subpopulations in adipose tissue undergo specific metabolic alterations, including enhanced glycolysis, mitochondrial dysfunction, and lipid metabolic remodeling, which directly influence their functional polarization and inflammatory responses [43,46,47,48]. In addition, emerging evidence suggests that extracellular vesicles and adipose tissue senescence-associated secretory phenotype (SASP) play key roles in mediating adipocyte-immune cell-to-cell communication [44,49].

### 2.2. The Effects of Immune Disregulation on Obesity

While obesity clearly induced inflammatory responses, accumulating evidence suggested that inflammation itself is not merely a consequence of obesity but actively contributes to its development and maintenance, creating a bidirectional relationship that perpetuated metabolic dysfunction. This section examines how inflammation promotes and sustains obesity through multiple mechanisms.

Inflammatory signaling directly influenced adipose tissue function and energy homeostasis. Activation of inflammatory pathways, particularly those involving inhibitor of nuclear factor kappa-B kinase subunit beta/nuclear factor kappa B (IKKβ/NF-κB) and c-Jun N-terminal kinase (JNK), has been shown to interfere with insulin signaling and adipocyte function [50,51]. While initially thought to directly promote adiposity, the relationship is more complex. In transgenic mouse models, the hepatic activation of NF-κB pathway leads to systemic insulin resistance; however, tissue-specific effects vary considerably [52]. Proinflammatory cytokines such as TNF-α and IL-1β have been demonstrated to alter adipocyte metabolism, primarily by inhibiting adipogenesis and impairing mitochondrial function and fatty acid oxidation [53,54]. Contrary to earlier assumptions, most studies indicate that TNF-α actually suppresses rather than enhances lipogenesis by downregulating adipogenic genes such as peroxisome proliferator-activated receptor gamma (PPAR-γ) and CCAAT/enhancer-binding protein alpha (C/EBPα) while promoting lipolysis [16]. IL-6, another key inflammatory mediator elevated in obesity, exhibits complex and sometimes contradictory effects on adiposity. While acute IL-6 signaling could promote energy expenditure through central mechanisms, chronic elevation of IL-6 has been associated with metabolic dysfunction, though its direct effects on adipocyte lipid metabolism remain debated [55,56].

A hallmark of obesity is the altered composition and function of immune cells within adipose tissue. In lean individuals, adipose tissue contains primarily M2-polarized macrophages, regulatory T cells (Tregs), and type 2 innate lymphoid cells (ILC2s), which collectively maintain an anti-inflammatory environment conducive to metabolic homeostasis [57,58]. With obesity progression, this immune profile shifts dramatically. Adipose tissue becomes infiltrated with CD8+ T cells and neutrophils, which precede and facilitate the recruitment of proinflammatory M1 macrophages [59,60]. These M1 macrophages form characteristic crown-like structures around dying adipocytes and secrete proinflammatory cytokines that further promote immune cell recruitment and inflammation [61]. Simultaneously, regulatory immune populations such as Tregs and eosinophils decrease, exacerbating the inflammatory imbalance [62,63]. Recent studies using single-cell RNA sequencing revealed even greater complexity in adipose tissue immune populations during obesity, identifying novel subsets of macrophages beyond the simple M1/M2 dichotomy. For example, lipid-associated macrophages (LAMs) expressing Trem2 have been identified as playing important roles in metabolic homeostasis [64]. Other research has defined additional macrophage populations with distinct functional profiles that evolve during obesity progression [65].

Inflammation extends beyond peripheral tissues in obesity, significantly affecting central nervous system regulation of energy balance. Hypothalamic inflammation, particularly in the arcuate nucleus, emerges early in diet-induced obesity and contributes to leptin and insulin resistance, disrupting the central regulation of food intake and energy expenditure [66,67]. High-fat diet consumption activates microglia and astrocytes in the hypothalamus, leading to local production of inflammatory cytokines that impairs the function of anorexigenic pro-opiomelanocortin (POMC) neurons while enhancing the activity of orexigenic Neuropeptide Y/Agouti-Related Peptide (NPY/AgRP) neurons [68]. Interestingly, this neuroinflammation can be observed as early as 24 h after high-fat diet initiation, preceding significant weight gain, suggesting it may be a cause rather than consequence of obesity [66]. This neuroinflammation creates a vicious cycle where impaired hypothalamic signaling promotes hyperphagia and reduces energy expenditure, further exacerbating weight gain and systemic inflammation [69].

**Figure 1:**
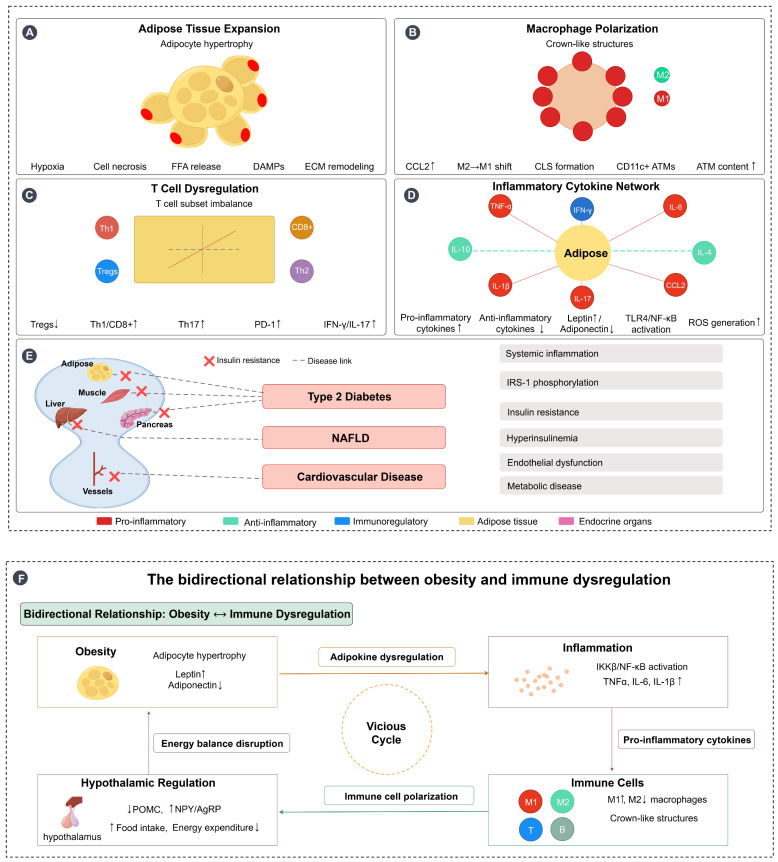
Immunometabolic interactions in obesity and bidirectional relationship with immune dysregulation. (**A**) Adipose tissue expansion: Obesity-induced adipocyte hypertrophy leads to tissue hypoxia, cell necrosis, free fatty acid (FFA) release, damage-associated molecular patterns (DAMPs) production, and extracellular matrix (ECM) remodeling, creating a proinflammatory microenvironment. (**B**) Macrophage polarization: Adipose tissue macrophages (ATMs) undergo polarization from the anti-inflammatory M2 phenotype to the proinflammatory M1 phenotype, with increased CCL2 expression facilitating macrophage recruitment and formation of crown-like structures (CLS) around dying adipocytes, characterized by CD11c+ ATMs accumulation. (**C**) T cell dysregulation: Obesity disrupts T cell subset balance, characterized by decreased regulatory T cells (Tregs↓) and increased proinflammatory T cell populations (Th1↑, CD8+↑, Th17↑), along with elevated PD-1 expression and increased production of inflammatory mediators (IFN-γ↑, IL-17↑). (**D**) Inflammatory cytokine network: Adipose tissue becomes the central hub of inflammatory signaling, with increased production of proinflammatory cytokines (TNF-α, IL-6, IL-1β, CCL2) and decreased anti-inflammatory cytokines (IL-10, IL-4). This is accompanied by altered adipokine secretion (leptin↑, adiponectin↓), TLR4/NF-κB pathway activation, and reactive oxygen species (ROS) generation. (**E**) Systemic inflammation and metabolic consequences: Obesity-induced chronic inflammation affects multiple target organs including muscle, liver, pancreas, and blood vessels, leading to insulin resistance and the development of metabolic diseases including type 2 diabetes, NAFLD, and cardiovascular disease through interconnected pathological pathways. (**F**) Bidirectional relationship forming a vicious cycle: Obesity and immune dysregulation create a self-perpetuating cycle where obesity promotes inflammation through adipokine dysregulation and tissue dysfunction, inflammation disrupts immune cell function and promotes further metabolic dysfunction, and hypothalamic inflammation impairs central energy regulation (POMC↓, NPY/AgRP↑), leading to increased food intake and decreased energy expenditure. This energy imbalance exacerbates obesity, completing the vicious cycle. Arrows: → indicate direct effects or pathways; ↔ indicate reciprocal interactions; symbols: ↑ indicates increased/upregulated; ↓ indicates decreased/downregulated. Figure designed by authors.

In summary, obesity triggers a series of immune dysregulations characterised by a chronic low-grade inflammatory state that disrupts immune homeostasis through a variety of mechanisms, including aberrant adipokine secretion, elevated levels of inflammatory cytokines, and alterations in the function of a wide range of immune cells. At the same time, inflammation itself is not solely a consequence of obesity, but is an active contributor to its development, creating a vicious cycle by affecting adipose tissue function, energy balance, and hypothalamic regulation. This complex interaction leads to increased susceptibility to infection and elevated risk of chronic disease, and understanding these mechanisms is critical to developing targeted therapeutic strategies to help improve obesity-related diseases and immunotherapy outcomes. Figure 1 illustrates these complex immunometabolic interactions in obesity, highlighting the bidirectional relationship between obesity and immune dysregulation, from adipose tissue expansion and immune cell polarization to systemic inflammation and the establishment of a self-perpetuating pathological cycle.

## 3. Gut Flora and Immune Regulation

The gut microbiome is increasingly recognized as a pivotal component in the regulation of immune responses and carries significant implications for the pathophysiology of obesity. Recent studies have underscored that obesity could lead to a reconfiguration of the microbial ecosystem along the gut–liver axis [70]. This transformation contributes to altered immune responses that foster systemic inflammation and metabolic dysfunction [71]. Specifically, research conducted by Galaris et al. demonstrated that the microbiome characteristic of obesity enhances energy extraction from dietary sources, which in turn promotes fat accumulation and aggravated obesity-related health conditions. Moreover, Santos-Paulo et al. spotlighted the therapeutic promise of interventions aimed at microbiome modulation, such as fecal microbiota transplantation (FMT) and dietary modifications, which show potential in ameliorating metabolic issues in individuals diagnosed with obesity [72]. Their analysis revealed that microbiome modulation approaches such as FMT from lean donors demonstrated significant improvements in peripheral insulin sensitivity and glycemic control (reduced HbA1c) in metabolic syndrome patients. Dietary interventions, particularly those rich in microbiota-accessible carbohydrates, could reshape gut microbial populations by increasing beneficial bacteria associated with metabolic health. The review also noted that specific probiotic strains (particularly *Lactobacillus gasseri*) showed efficacy in reducing abdominal visceral fat by 4.6% and waist circumference by 1.8% in clinical trials.

Examining the intricate relationship between gut flora and immune regulatory mechanisms reveals how the microbiome participates in the maintenance of metabolic health. Hossain et al. illustrated that obesity disrupts bile acid metabolism, which subsequently influences the farnesoid X receptor (FXR) in the liver [73]. This modulation could profoundly affect insulin sensitivity and immune responses within the body. Notably, specific gut bacteria, such as *Akkermansia muciniphila*, has been correlated with enhanced metabolic parameters and heightened insulin sensitivity, indicating that distinct microbial compositions might have offered protective effects against the development of obesity and its associated complications [74].

**Figure 2:**
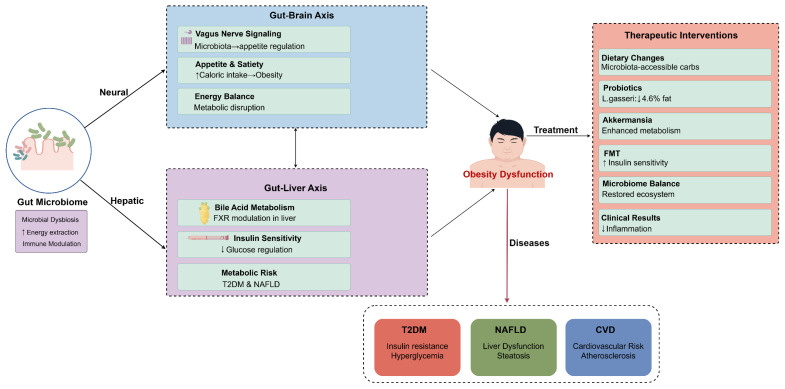
Multi-axial mechanisms of gut microbiota regulation of obesity and therapeutic intervention strategies. This figure illustrates key pathways through which gut microbiota influences metabolic health via neural and hepatic pathways. The gut–brain axis affects metabolic function through vagus nerve signaling, appetite regulation, and energy balance, while the gut–liver axis participates in metabolic risk regulation through bile acid metabolism, FXR regulation, and insulin sensitivity. Gut microbiota dysbiosis can lead to obesity dysfunction, which in turn triggers various metabolic diseases such as T2DM, NAFLD, and CVD. Right side of the figure presents therapeutic intervention strategies targeting gut microbiota, including dietary modifications, probiotic supplementation, *Akkermansia*, fecal microbiota transplantation, and microbiota balancing. These interventions can reduce inflammatory responses and improve metabolic outcomes. Figure designed by authors.

The role of the gut microbiome further extended to the regulation of appetite and satiety through pathways linked to the gut–brain axis. Research by Longo et al. explored the intricate relationships among the vagus nerve, gut microbiota, and metabolic regulation, underscoring the potential for dietary interventions and the use of probiotics to rehabilitate a balanced gut microbiome [75]. The interconnectedness of these pathways exemplifies the multifaceted mechanisms by which gut microbiota influence not only obesity and metabolic dysfunction but also immune responses. This intricate interplay highlights the necessity for further research in understanding the gut microbiome’s role in metabolic health, as summarized in Figure 2. It is worth noting that metabolic abnormalities have reached epidemic levels, and the complex and dynamic nature of the gut microbiota, a key player in its pathophysiology, could initiate a range of physiological processes and disruptions to the microbiome that could have a detrimental effect on overall health [76].

## 4. Immunomodulation in Metabolic Diseases

The role of immune regulation in obesity-related metabolic diseases encompasses various conditions, notably T2DM, NAFLD, dyslipidemia, hypertension, CVD, polycystic ovary syndrome (PCOS), chronic kidney disease (CKD), and obstructive sleep apnea (OSA). The complex interplay between chronic inflammation, immune dysfunction, and metabolic processes is pivotal in the pathogenesis of these diseases.

In T2DM, the chronic state of low-grade inflammation is intimately linked to insulin resistance. Immune cells, particularly macrophages, produce proinflammatory cytokines that disrupt insulin signaling pathways, thereby exacerbating metabolic dysregulation [77]. The release of these cytokines is often reflective of an imbalance in T cell subsets, which could be influenced by obesity-related factors [78]. Indeed, immunosenescent T cells have been associated with heightened inflammation in hypertensive states and forms of metabolic dysfunction [79].

Dyslipidemia, commonly associated with the metabolic syndrome, has been linked to immune dysregulation. Although Chen et al. did not directly address dyslipidemia, their discussion of the metabolic syndrome and its relationship to systemic inflammation suggests that targeting inflammatory pathways might help manage lipid profiles and reduce cardiovascular risk [80]. The role of immune mechanisms in hypertension has also come under scrutiny, with recent findings suggesting that inflammation leads to vascular dysfunction, which in turn triggers hypertension [81]. This highlights the potential for immunomodulatory therapies to alleviate hypertension by targeting underlying inflammatory processes.

NAFLD is characterized by an abnormal immune response that leads to hepatic inflammation and fibrogenesis, where immune cells infiltrate liver tissues in response to lipotoxicity and other metabolic disruptions [82]. The activation of these immune cells contributes further to the steatotic condition, which is commonly seen in obese individuals [83]. Chronic inflammation and dysregulated immune signaling pathways play crucial roles in hepatic insulin resistance and lipid metabolism disturbances, reinforcing the connection between obesity and NAFLD [84].

Hypertension, often correlated with obesity, provides another perspective on immune dysregulation. Hypertensive conditions have been noted to involve the activation of innate immune responses that, alongside neuroimmune communications, contribute to the regulation of blood pressure [85]. Specifically, T lymphocytes have been implicated in modulating vascular tone and inflammatory responses, suggesting that targeting immune pathways could offer new therapeutic strategies for managing hypertension [86]. Furthermore, myeloid-derived suppressor cells have been shown to exert regulatory effects on blood pressure, providing further evidence of the intricate link between the immune system and cardiovascular health [87].

In the context of PCOS, the intersection of hormonal imbalance and immune dysfunction is critical. Women with PCOS display a chronic inflammatory state that is linked to insulin resistance and features of metabolic syndrome [88]. Dysregulation of immune responses has also been observed alongside variations in the levels of androgens, establishing a feedback loop where immune dysfunction exacerbates both metabolic and reproductive issues [89]. Moreover, altered T cell activity and the presence of myeloid-derived suppressor cells has been documented in PCOS, suggesting a significant role for immune cells in the pathophysiology of this condition [90].

CKD also illustrates the impact of immune regulation in obesity-related diseases. In CKD, inflammation often correlates with renal injury, which can be exacerbated by obesity-induced immune cell activation [91]. The relevance of immune dysregulation in CKD is underscored by the promotion of vascular inflammation, which further complicates hypertension and cardiovascular complications in these patients [92].

Lastly, OSA, though less frequently discussed in the context of immune regulation, also shares common pathways with obesity and inflammation. The intermittent hypoxia associated with OSA can induce a systemic inflammatory response, altering immune function and potentially leading to further metabolic complications, including T2DM and cardiovascular issues [93].

**Figure 3:**
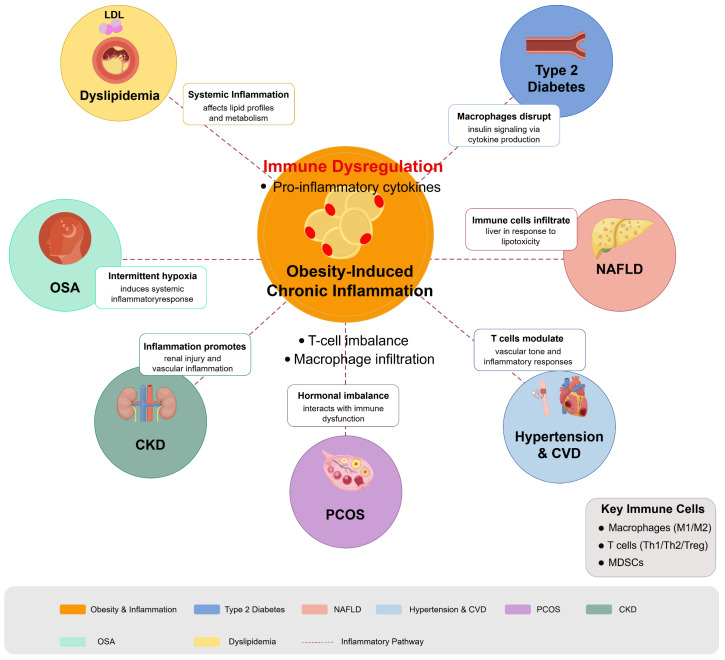
Immunomodulation in obesity-related metabolic diseases. Diagram illustrates how obesity-induced chronic inflammation serves as a central driver for multiple metabolic disorders through immune dysregulation pathways. Obesity triggers adipose tissue inflammation characterized by macrophage infiltration, T cell imbalance, and proinflammatory cytokine production, which radiates outward to impact multiple organ systems. Key conditions affected include T2DM (insulin signaling disruption), NAFLD (liver lipotoxicity and immune cell infiltration), dyslipidemia (altered lipid metabolism), hypertension and cardiovascular disease (vascular inflammation), PCOS (hormonal-immune interactions), CKD (renal inflammation), and OSA (intermittent hypoxia-induced inflammation). Figure designed by authors.

Overall, the regulation of immune responses plays a fundamental role in various obesity-related metabolic diseases, highlighting the potential for therapeutic strategies that target immune pathways. Continued research into these interactions is crucial for developing targeted interventions that address both the metabolic and inflammatory components of these multifaceted conditions. Figure 3 illustrates how obesity-induced chronic inflammation acts as a hub connecting multiple metabolic conditions through shared inflammatory pathways. Table 2 provides a systematic summary of the immune mechanisms of various obesity-related metabolic diseases and their potential immunomodulatory therapeutic approaches, which provides a comprehensive reference framework for clinical decision-making and future research directions.

## 5. Immunomodulation as a Novel Target for Obesity Treatment

The investigation of immunomodulation as a novel target for obesity treatment is rapidly evolving, revealing the intricate relationship between immune system dysfunction and metabolic disorders. Current research indicates that immune dysregulation is a hallmark of obesity, characterized by chronic low-grade inflammation and disrupted immune cell function that both drives obesity pathogenesis and complicates management of related comorbidities, including diabetes and cancer [102]. While chronic inflammation contributes significantly to these complications, it is crucial to recognize the variability in inflammatory profiles among individuals with obesity, highlighting the multifactorial nature of this complex disease influenced by genetic, behavioral, hormonal, and environmental factors. Recent findings underscore the critical role of specific immune cell subsets in adipose tissue inflammation, with studies by Liu et al. (2018) demonstrating how obesity alters T cell function and how different T cell subsets can either worsen or alleviate inflammation within adipose tissue [5]. Similarly, Piening et al. (2024) established that obesity-associated T cell dysfunction impairs immunosurveillance and increases cancer risk, emphasizing the need for comprehensive immunomodulatory strategies that address broader aspects of immune dysfunction [34].

Beyond immune cell dynamics, recent research has illuminated the role of adipokines, particularly leptin, in modulating immune responses, with Vick et al. (2023) noting that elevated leptin levels during diet-induced obesity might reduce the efficacy of certain cancer immunotherapies [103]. Additional pathways, such as inhibition of the IKKβ/NF-κB signaling cascade, offer promising therapeutic targets for ameliorating obesity-induced inflammation [104]. The “obesity paradox”—where moderate obesity might correlate with improved outcomes in specific cancer treatments while severe obesity predicts poorer prognoses—further complicates our understanding [105]. This paradoxical observation highlights the complexity of immune responses in obesity and their implications for immunotherapy efficacy, suggesting that a deeper understanding of obesity’s effects on immune mechanisms could lead to more tailored treatment strategies for patients with both obesity and cancer.

Furthermore, innovative approaches such as the use of nanotechnology for immunotherapy are being explored. Yan et al. proposed a method using gold nanobipyramids to modulate macrophage activity in adipose tissue, thereby enhancing the clearance of excess adipocytes and potentially improving metabolic health [106]. This represents a novel intersection of immunology and nanomedicine in the fight against obesity.

Fecal microbiota transplantation (FMT), an innovative therapeutic strategy for microbiome modulation, has shown great potential in the treatment of obesity [107]. Clinical studies have shown that transplantation of gut microbes from lean individuals could significantly improve metabolic markers, modulate immune function, and potentially attenuate obesity-related chronic inflammatory responses [108]. Specifically, by remodeling gut microbiota diversity, FMT could directly influence host metabolic processes, including improving insulin sensitivity, modulating lipid metabolism, and attenuating systemic inflammation [109]. Although FMT has shown encouraging promise in the treatment of obesity, large-scale clinical studies are needed to validate its long-term efficacy and safety, especially in terms of individualized response in different populations.

The current cutting-edge literature indicates a growing understanding of the role of the immune system in obesity, and immune modulation is seen as an emerging and promising target for the treatment of obesity. By gaining a deeper understanding of the complex interactions between immune cells, adipokines, and metabolic pathways, researchers are paving the way for the development of innovative therapeutic strategies that are expected to significantly improve the prognosis of obese patients and their comorbidities. Table 3 summarizes a comparative analysis of the current major immunomodulatory therapeutic strategies, including their mechanism of action, clinical or experimental evidence, strengths and limitations, and potential synergies with existing treatments, providing a comprehensive frame of reference for clinical decision-making and future research directions. Due to the heterogeneity of inflammatory profiles, genetic factors, and comorbidities in obese patients, optimal immunomodulatory strategies vary from individual to individual. Artificial intelligence approaches help to identify patients who are best suited to receive specific immunomodulatory interventions, thereby supporting more personalized treatment regimens. Available evidence suggests that combination therapeutic regimens that simultaneously target inflammatory and metabolic components of obesity might provide superior therapeutic outcomes over single-target therapies [110,111].

To better understand the application of immunomodulatory treatment strategies in clinical practice, we searched the WHO International Clinical Trials Registry Platform (ICTRP) database (https://trialsearch.who.int/Default.aspx) for the latest studies. Table 4 summarizes the main clinical trials registered in 2025 related to immunomodulatory therapy for obesity. These trials reflect a wide range of treatment modalities, from traditional herbal medicines (such as puerarin) to modern biopharmaceuticals (such as GLP-1 receptor agonists), as well as diverse strategies ranging from single interventions to combination therapies. Notably, these clinical studies demonstrate the emerging trend of immunomodulatory therapy in the translational application of obesity treatment. For example, the NCT06968208 trial explored the potential of puerarin to achieve weight loss by regulating inflammatory factors, while the NCT06863363 study on the combination therapy of PEG-rhGH and semaglutide represented a new strategy of multi-pathway synergistic regulation. These clinical studies not only enriched the evidence base for immunomodulatory therapy in obesity but also provided important references for future personalized precision treatment.

## 6. Future Research Directions and Challenges in Obesity and Immunomodulation

The ongoing exploration of immunomodulatory treatment strategies focused on the inflammatory state of obesity underscores the complexity and multifactorial nature of this condition. Chronic low-grade inflammation (CLGI) is indeed a hallmark of obesity, but it is crucial to recognize that not all individuals with obesity present the same inflammatory profiles. Factors such as genetic predispositions, behavioral patterns, hormonal influences, and environmental conditions can significantly impact the immune response and overall health outcomes of obese patients [122,123]. Thus, while inflammation-targeted therapies could promise benefits, a more nuanced understanding and approach are likely required for effective obesity management.

The complexity of obesity necessitates comprehensive treatment strategies that integrate immunomodulatory therapies with existing pharmacotherapies, such as metformin and GLP-1 receptor agonists, known for their efficacy in managing obesity and its metabolic consequences [124,125]. Studies indicate that combining these therapies could potentially enhance treatment outcomes by addressing not only inflammation but also other underlying biological and sociological factors related to obesity [126,127]. For instance, the obesity paradox—where increased adiposity correlates with better outcomes in certain immunotherapy contexts—reveals a need for tailored treatment regimens rather than a one-size-fits-all approach [128,129]. Such combinations could mitigate the side effects associated with immunotherapy—namely, the heightened risk of infections due to immunosuppressive agents like tumor necrosis factor alpha (TNF-α) and interleukin 1 beta (IL-1β) inhibitors [130,131].

Immunotherapies can lead to significant adverse effects, particularly immunosuppression, which may elevate the risk of opportunistic infections, as evidenced by studies linking TNF-α inhibitors with increased susceptibility to infections [132]. Conversely, while IL-1β inhibitors might pose fewer risks, their clinical efficacy remains variable [133]. As such, personalized treatment plans that incorporate patients’ unique immune profiles and metabolic conditions are essential. Ongoing research into immunophenotyping may offer valuable insights for developing these personalized strategies, ultimately improving therapeutic outcomes and minimizing adverse effects.

**Figure 4:**
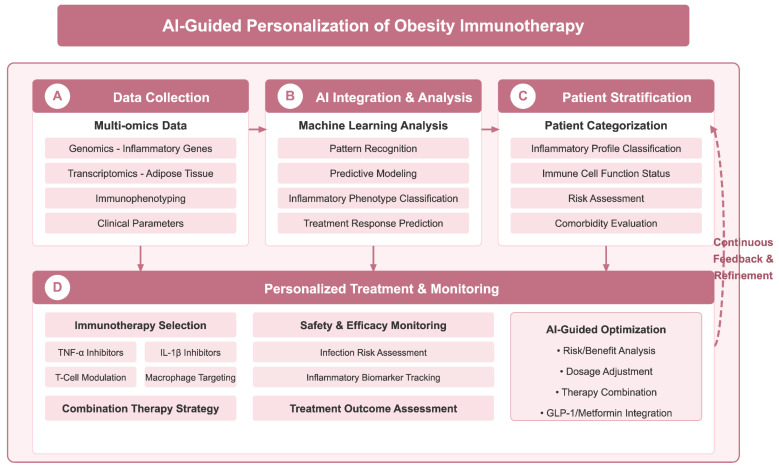
AI-assisted personalized decision-making framework for obesity immunotherapy. Framework achieves precision therapy through four key stages: (**A**) data collection stage, which integrates multi-omics data such as genomics, transcriptomics, immunophenotyping, and clinical parameters; (**B**) AI integration and analysis stage, which leverages machine learning for pattern recognition, predictive modelling, classification of inflammatory phenotypes, and prediction of therapeutic response; (**C**) patient stratification stage, which provides individual classification based on inflammation profiles, immune cell functional status, risk assessment, and comorbidity assessment; and (**D**) personalized treatment and monitoring phase, guiding immunotherapy selection (e.g., TNF-α inhibitors, IL-1β inhibitors, etc.), safety monitoring and treatment efficacy assessment, and AI-guided risk-benefit analysis, dosage adjustment, and optimization of combination therapy strategies. A continuous feedback and optimization mechanism (dotted line on the right) ensures that treatment regimens are dynamically adjusted according to patient response. Figure designed by authors.

Furthermore, the integration of artificial intelligence (AI) into the design and application of immunotherapies holds potential for optimizing patient selection for treatments. AI models could analyze extensive datasets to discern patterns in how specific immune responses correlate with treatment success, potentially identifying subsets of patients more likely to derive benefit from targeted therapies [134,135]. This predictive modeling could significantly enhance the effectiveness of personalized immunotherapy regimens and inform the development of future pharmacological approaches. With the deepening understanding of obesity-related immune disorders, AI technologies offer new possibilities for the design of personalized immunotherapy regimens; as shown in Figure 4, an AI-assisted decision-making framework integrating multi-omics data, patient stratification, and treatment optimization may significantly improve the precision of obesity immunotherapy. Notably, microRNAs (miRNAs) show great potential in the diagnosis of non-alcoholic steatohepatitis (NASH), dyslipidemia, obesity, and their associated metabolic diseases, where they can not only serve as potential biomarkers, but also provide key molecular insights into disease progression and therapeutic response [136].

In summary, while immunomodulatory therapies continue to exhibit significant potential in obesity treatment and associated metabolic disorders, their implementation must be approached carefully, considering safety, efficacy, and the need for personalized care. As researchers delve into the interplay of obesity, inflammation, and cancer, bridging the gap between clinical practice and emerging therapeutic strategies will be crucial for advancing patient care in this complex and burgeoning field.

## 7. Conclusions

Immunomodulatory strategies represent promising approaches for obesity management, including anti-inflammatory agents, microbiome interventions, and immune cell-targeted therapies. However, challenges remain, particularly regarding immunotherapy side effects and the need for personalized treatment approaches. Future research should focus on developing precision immunotherapies utilizing artificial intelligence to optimize patient selection and treatment protocols. A deeper understanding of obesity’s immunometabolic mechanisms will facilitate the development of innovative therapeutic strategies that address both the inflammatory and metabolic components of this complex condition, ultimately improving patient outcomes and reducing the global burden of obesity-related diseases.

## Figures and Tables

**Table 1:** Role of immune cells in obesity.

Immune Cell Types	Mechanisms	Changes in Obesity Status	Influences	Study Type	References
Macrophages	Mediates adipose tissue inflammation	M1 proinflammatory phenotype ↑, M2 anti-inflammatory phenotype ↓	Chronic inflammation and insulin resistance	In vitro studies, animal studies, and clinical studies	[36]
T Cells	Modulating the immune response	Impaired CD4+ and CD8+ T cell function, ↑ PD-1 receptor expression	Immune imbalance and reduced T cell function and numbers	In vitro studies, animal studies, and clinical studies	[37,38,39]
B Cells	Produces antibodies, regulates T cells	Abnormal antibody production and imbalance in T cell regulation	Chronic inflammation	In vitro studies, animal studies, and clinical studies	[40]
NK Cells	Tumour surveillance and antiviral	Functional decline	Promoting inflammation	In vitro studies, animal studies, and clinical studies	[41]
Dendritic Cells	Antigen presentation and T cell activation	Abnormal antigen presentation, inflammatory signal release	Tumour immunity and reduced antiviral capacity	In vitro studies, animal studies, and clinical studies	[42]

Abbreviations: PD-1, programmed cell death protein 1; CD4+, cluster of differentiation 4 positive; CD8+, cluster of differentiation 8 positive. Symbols: ↑, increased/upregulated; ↓, decreased/downregulated.

**Table 2:** Obesity-related metabolic diseases: immune mechanisms and potential therapies *.

Disease	Immune Mechanism	Potential Immunomodulation	Study type	References
T2DM	Inflammation → Insulin resistance	Sodium-glucose cotransporter 2 (SGLT2) inhibitors improve insulin sensitivity	In vitro studies, animal studies, and clinical studies	[94]
NAFLD	Inflammatory cytokines → Liver fat accumulation and fibrosis	SGLT2 inhibitors reduce liver fat and inflammation	In vitro studies and clinical studies	[95]
Dyslipidemia	Inflammation → Lipid imbalance (↑LDL, ↓HDL)	Anti-inflammatory therapy may improve lipid levels	Clinical study	[96]
Hypertension	Inflammation → Vascular dysfunction	Targeting inflammation may lower blood pressure	In vitro studies, animal studies, and clinical	[97]
CVD	Chronic inflammation → Atherosclerosis	Immune modulation may reduce heart disease risk	In vitro studies, animal studies, and clinical studies	[98]
PCOS	Inflammation → Insulin resistance and hormonal imbalance	Immunotherapy may regulate metabolism and reproduction	In vitro studies, animal studies, and clinical studies	[99]
CKD	Inflammation → Kidney damage	Anti-inflammatory strategies may slow CKD progression	Clinical studies and animal studies	[100]
OSA	Inflammatory response→ Worsened insulin resistance and CVD risk	Inflammation control may improve OSA-related outcomes	In vitro studies and animal studies	[101]

* All abbreviations are defined in the main text and abbreviations list. Symbols: ↑, increased; ↓, decreased.

**Table 3:** Immunomodulatory treatment strategies for obesity.

Comparative Analysis of Mechanisms, Evidence, Advantages, Limitations, and Synergistic Potential							
	Mechanism of Action	Clinical/Experimental Evidence	Advantages	Limitations	Potential Synergies	Study Type	References
Cytokine Inhibitors (TNF-α, IL-1β)	Neutralize proinflammatory cytokines elevated in obesity-related inflammation	TNF-α inhibitors show efficacy in inflammatory diseases but variable results in metabolic improvement	Direct targeting of key inflammatory mediators; established safety profile from other indications	Increased infection risk; immunosuppressive effects; possible metabolic side effects	GLP-1 receptor agonists; metformin	Animal study (IL-1β inhibitors); clinical study (TNF-α inhibitors)	[112,113]
T Cell-Targeted Approaches	Modulate T cell subpopulations to restore balance between pro- and anti-inflammatory responses	T cell dysregulation directly linked to obesity-related inflammation and metabolic dysfunction	Highly specific targeting of key immune regulators; addresses root immune pathology	Complex T cell subset interactions; potential for autoimmune complications	Anti-inflammatory agents; microbiome modulators	Animal study	[114,115]
Macrophage Polarization Strategies	Shift adipose tissue macrophages from proinflammatory M1 to anti-inflammatory M2 phenotype	M1 macrophage infiltration in adipose tissue correlates with insulin resistance and metabolic dysfunction	Targets primary cellular drivers of adipose inflammation; potential for tissue-specific effects	Difficulty achieving tissue specificity; potential immunosuppressive effects	Insulin sensitizers; anti-inflammatory treatments	In vitro study and animal study	[116]
Adipokine Modulators (e.g., Leptin Antagonists)	Regulate adipokine signaling to reduce proinflammatory effects and improve metabolic signaling	Elevated leptin levels in obesity promote inflammation and impair immunotherapy responses	Targets intersection between metabolism and immunity; addresses obesity-specific pathophysiology	Complex pleiotropic effects; potential impact on appetite regulation and energy homeostasis	Cancer immunotherapies; metabolic regulators	Animal study and clinical study	[117]
NF-κB Pathway Inhibitors	Inhibit IKKβ/NF-κB signaling cascade that mediates inflammatory responses in multiple tissues	NF-κB activation central to obesity-induced inflammation and insulin resistance	Targets key upstream inflammatory mediator; potential for broad anti-inflammatory effects	Risk of broad immunosuppression; potential toxicity due to ubiquitous NF-κB functions	Insulin sensitizers; targeted immunotherapies	Animal studies, in vitro studies and a small number of clinical studies	[104]
Nanomedicine Approaches	Use engineered nanoparticles (e.g., gold nanobipyramids) to modulate macrophage function and enhance adipocyte clearance	Gold nanobipyramids demonstrated to promote adipocyte clearance via macrophage modulation in experimental models	Highly targeted approach; potential for reduced systemic effects; innovative delivery mechanisms	Early stage of development; unknown long-term safety profile; manufacturing complexity	Conventional weight loss therapies; metabolic regulators	In vitro study and animal study	[106]
Microbiome-Based Interventions	Modulate gut microbiota to reduce systemic inflammation and improve metabolic signaling	Gut microbiome alterations linked to obesity-related inflammation; specific bacteria (e.g., *Akkermansia*) show metabolic benefits	Non-invasive; potentially addresses root causes; favorable safety profile	Inter-individual variability; complex microbiome interactions; durability of effects	Dietary interventions; anti-inflammatory agents	Animal studies, in vitro studies, and clinical studies	[118,119]
Combination Immunometabolic Therapies	Integrate immunomodulatory approaches with metabolic regulators (e.g., GLP-1 agonists) for synergistic effects	Emerging evidence that combined targeting of inflammation and metabolism provides superior outcomes	Addresses multiple pathogenic mechanisms; personalization potential; improved efficacy	Increased potential for adverse effects; complex drug interactions; higher treatment costs	AI-guided treatment selection; personalized medicine approaches	Animal studies, in vitro studies, and clinical studies	[120,121]

Abbreviations: GLP-1, glucagon-like peptide 1.

**Table 4:** Clinical trials related to obesity immunomodulatory therapy registered on WHO platform by 2025.

Trial ID	Public Title	Date of Registration	Study Type
NCT06968208	Randomized Controlled Trial of Puerarin for Obesity Treatment	5 May 2025	Interventional
CTRI/2025/04/085671	Obesity treated with Ashuwathi chooranam and siddhar yoga therapy	25 April 2025	Interventional
NCT06974851	A Phase III Study Evaluating the Efficacy and Safety of HRS9531 Injection in Subjects With Obstructive Sleep Apnea (OSA) and Obesity	24 April 2025	Interventional
ChiCTR2500100289	A Multicenter, Randomized, Open-label, Parallel-controlled Phase III Study Comparing the Efficacy and Safety of Semaglutide Injection and Wegovy^®^ for Weight Loss in Obese Subjects	7 April 2025	Interventional
NCT06863363	PEG-rhGH and Semaglutide Combination Therapy in Non-Diabetic Obese Adults	28 February 2025	Interventional
NCT06874270	Metabolic Phenotyping for Personalized Obesity Therapy	27 February 2025	Observational
NCT06809166	Metabolic and Behavioural Effects of CONTRAVE as Potential Mechanisms of Weight Loss in Adults With Obesity	22 January 2025	Interventional
NCT06761703	A Study to Determine the Feasibility of Online Recruitment of People Using an Anti-Obesity Medication for Weight Loss	1 January 2025	Observational

## Abbreviations

T2DM, type 2 diabetes mellitus; CVD, cardiovascular disease; NAFLD, non-alcoholic fatty liver disease; TNF-α, tumor necrosis factor-alpha; IL-6, interleukin 6; IL-1β, interleukin 1 beta; IL-33, interleukin 33; IL-17, interleukin 17; IL-10, interleukin 10; IL-4, interleukin 4; CRP, C-reactive protein; PD-1, programmed cell death protein 1; IFN-γ, interferon gamma; Tregs, regulatory T cells; NK cells, natural killer cells; ATMs, adipose tissue macrophages; IKKβ, inhibitor of nuclear factor kappa-B kinase subunit beta; NF-κB, nuclear factor kappa B; JNK, c-Jun N-terminal kinase; PPAR-γ, peroxisome proliferator-activated receptor gamma; C/EBPα, CCAAT/enhancer-binding protein alpha; POMC, pro-opiomelanocortin; NPY/AgRP, neuropeptide Y/agouti-related peptide; WAT, white adipose tissue; BAT, brown adipose tissue; ILC2s, type 2 innate lymphoid cells; LAMs, lipid-associated macrophages; FMT, fecal microbiota transplantation; FXR, farnesoid X receptor; PCOS, polycystic ovary syndrome; CKD, chronic kidney disease; OSA, obstructive sleep apnea; SGLT2, sodium-glucose cotransporter 2; LDL, low-density lipoprotein; HDL, high-density lipoprotein; GLP-1, glucagon-like peptide 1; CLGI, chronic low-grade inflammation; SASP, senescence-associated secretory phenotype; AI, artificial intelligence; TLR4, toll-like receptor 4; ROS, reactive oxygen species; FFA, free fatty acid; DAMPs, damage-associated molecular patterns; ECM, extracellular matrix; CCL2, C-C motif chemokine ligand 2; CLS, crown-like structures; M1, classically activated macrophages; M2, alternatively activated macrophages; HbA1c, hemoglobin A1c; WHO, World Health Organization; ICTRP, International Clinical Trials Registry Platform; PEG-rhGH, pegylated recombinant human growth hormone; miRNAs, microRNAs; NASH, non-alcoholic steatohepatitis.

## References

[1] Lin K., Cheng W., Shen Q., Wang H., Wang R., Guo S., Wu X., Wu W., Chen P., Wang Y. (2023). Lipid Profiling Reveals Lipidomic Signatures of Weight Loss Interventions. Nutrients.

[2] Mohajan D., Mohajan H.K. (2023). Obesity and its related diseases: A new escalating alarming in global health. J. Innov. Med. Res..

[3] Mitra S., De A., Chowdhury A. (2020). Epidemiology of non-alcoholic and alcoholic fatty liver diseases. Transl. Gastroenterol. Hepatol..

[4] Lin X., Li H. (2021). Obesity: Epidemiology, pathophysiology, and therapeutics. Front. Endocrinol..

[5] Liu R., Nikolajczyk B.S. (2019). Tissue immune cells fuel obesity-associated inflammation in adipose tissue and beyond. Front. Immunol..

[6] Wu H., Ballantyne C.M. (2020). Metabolic inflammation and insulin resistance in obesity. Circ. Res..

[7] Yao J., Wu D., Qiu Y. (2022). Adipose tissue macrophage in obesity-associated metabolic diseases. Front. Immunol..

[8] Skuratovskaia D., Vulf M., Khaziakhmatova O., Malashchenko V., Komar A., Shunkin E., Shupletsova V., Goncharov A., Urazova O., Litvinova L. (2020). Tissue-specific role of macrophages in noninfectious inflammatory disorders. Biomedicines.

[9] Van der Zalm I., Van Der Valk E., Wester V., Nagtzaam N., Van Rossum E., Leenen P., Dik W. (2020). Obesity-associated T-cell and macrophage activation improve partly after a lifestyle intervention. Int. J. Obes..

[10] Taranto D., Kloosterman D.J., Akkari L. (2024). Macrophages and T cells in metabolic disorder-associated cancers. Nat. Rev. Cancer.

[11] Guglielmi V., Colangeli L., D’Adamo M., Sbraccia P. (2021). Susceptibility and Severity of Viral Infections in Obesity: Lessons from Influenza to COVID-19. Does Leptin Play a Role?. Int. J. Mol. Sci..

[12] Han V.X., Patel S., Jones H.F., Nielsen T.C., Mohammad S.S., Hofer M.J., Gold W., Brilot F., Lain S.J., Nassar N. (2021). Maternal acute and chronic inflammation in pregnancy is associated with common neurodevelopmental disorders: A systematic review. Transl. Psychiatry.

[13] Bell H.N., Zou W. (2024). Beyond the Barrier: Unraveling the Mechanisms of Immunotherapy Resistance. Annu. Rev. Immunol..

[14] Lasbleiz A., Gaborit B., Soghomonian A., Bartoli A., Ancel P., Jacquier A., Dutour A. (2021). COVID-19 and Obesity: Role of Ectopic Visceral and Epicardial Adipose Tissues in Myocardial Injury. Front. Endocrinol..

[15] Russo C., Maugeri A., Musumeci L., De Sarro G., Cirmi S., Navarra M. (2023). Inflammation and Obesity: The Pharmacological Role of Flavonoids in the Zebrafish Model. Int. J. Mol. Sci..

[16] Maurya R., Sebastian P., Namdeo M., Devender M., Gertler A. (2021). COVID-19 Severity in Obesity: Leptin and Inflammatory Cytokine Interplay in the Link Between High Morbidity and Mortality. Front. Immunol..

[17] Aghili S.M.M., Ebrahimpur M., Arjmand B., Shadman Z., Pejman Sani M., Qorbani M., Larijani B., Payab M. (2021). Obesity in COVID-19 era, implications for mechanisms, comorbidities, and prognosis: A review and meta-analysis. Int. J. Obes..

[18] Rohm T.V., Meier D.T., Olefsky J.M., Donath M.Y. (2022). Inflammation in obesity, diabetes, and related disorders. Immunity.

[19] Zha Z., Cheng Y., Cao L., Qian Y., Liu X., Guo Y., Wang J. (2021). Monomeric CRP Aggravates Myocardial Injury After Myocardial Infarction by Polarizing the Macrophage to Pro-Inflammatory Phenotype Through JNK Signaling Pathway. J. Inflamm. Res..

[20] Pérez-Pérez A., Vilariño-García T., Fernández-Riejos P., Martín-González J., Segura-Egea J.J., Sánchez-Margalet V. (2017). Role of leptin as a link between metabolism and the immune system. Cytokine Growth Factor Rev..

[21] Kiernan K., MacIver N.J. (2020). The Role of the Adipokine Leptin in Immune Cell Function in Health and Disease. Front. Immunol..

[22] He W., Wang H., Yang G., Zhu L., Liu X. (2024). The Role of Chemokines in Obesity and Exercise-Induced Weight Loss. Biomolecules.

[23] Zamarron B.F., Mergian T.A., Cho K.W., Martinez-Santibanez G., Luan D., Singer K., DelProposto J.L., Geletka L.M., Muir L.A., Lumeng C.N. (2017). Macrophage Proliferation Sustains Adipose Tissue Inflammation in Formerly Obese Mice. Diabetes.

[24] Li B., Sun S., Li J.-J., Yuan J.-P., Sun S.-R., Wu Q. (2023). Adipose tissue macrophages: Implications for obesity-associated cancer. Mil. Med. Res..

[25] Kawai T., Autieri M.V., Scalia R. (2021). Adipose tissue inflammation and metabolic dysfunction in obesity. Am. J. Physiol. Cell Physiol..

[26] Honce R., Schultz-Cherry S. (2019). Impact of Obesity on Influenza A Virus Pathogenesis, Immune Response, and Evolution. Front. Immunol..

[27] Almond M., Farne H.A., Jackson M.M., Jha A., Katsoulis O., Pitts O., Tunstall T., Regis E., Dunning J., Byrne A.J. (2023). Obesity dysregulates the pulmonary antiviral immune response. Nat. Commun..

[28] Sanchez-Infantes D., Stephens J.M. (2020). Adipocyte Oncostatin Receptor Regulates Adipose Tissue Homeostasis and Inflammation. Front. Immunol..

[29] Frasca D., Blomberg B.B. (2017). Adipose Tissue Inflammation Induces B Cell Inflammation and Decreases B Cell Function in Aging. Front. Immunol..

[30] Croce S., Avanzini M.A., Regalbuto C., Cordaro E., Vinci F., Zuccotti G., Calcaterra V. (2021). Adipose Tissue Immunomodulation and Treg/Th17 Imbalance in the Impaired Glucose Metabolism of Children with Obesity. Children.

[31] Kowitt C., Zhang Q. (2024). Interleukin-33 and Obesity-Related Inflammation and Cancer. Encyclopedia.

[32] Jin X., Qiu T., Li L., Yu R., Chen X., Li C., Proud C.G., Jiang T. (2023). Pathophysiology of obesity and its associated diseases. Acta Pharm. Sin. B.

[33] Zheng D., Liwinski T., Elinav E. (2020). Interaction between microbiota and immunity in health and disease. Cell Res..

[34] Piening A., Ebert E., Gottlieb C., Khojandi N., Kuehm L.M., Hoft S.G., Pyles K.D., McCommis K.S., DiPaolo R.J., Ferris S.T. (2024). Obesity-related T cell dysfunction impairs immunosurveillance and increases cancer risk. Nat. Commun..

[35] Dousdampanis P., Aggeletopoulou I., Mouzaki A. (2024). The role of M1/M2 macrophage polarization in the pathogenesis of obesity-related kidney disease and related pathologies. Front. Immunol..

[36] Liang W., Qi Y., Yi H., Mao C., Meng Q., Wang H., Zheng C. (2022). The Roles of Adipose Tissue Macrophages in Human Disease. Front. Immunol..

[37] Rebeles J., Green W.D., Alwarawrah Y., Nichols A.G., Eisner W., Danzaki K., MacIver N.J., Beck M.A. (2019). Obesity-Induced Changes in T-Cell Metabolism Are Associated with Impaired Memory T-Cell Response to Influenza and Are Not Reversed with Weight Loss. J. Infect. Dis..

[38] Wang Z., Aguilar E.G., Luna J.I., Dunai C., Khuat L.T., Le C.T., Mirsoian A., Minnar C.M., Stoffel K.M., Sturgill I.R. (2019). Paradoxical effects of obesity on T cell function during tumor progression and PD-1 checkpoint blockade. Nat. Med..

[39] Liu L., Hu J., Wang Y., Lei H., Xu D. (2021). The role and research progress of the balance and interaction between regulatory T cells and other immune cells in obesity with insulin resistance. Adipocyte.

[40] Oleinika K., Slisere B., Catalán D., Rosser E.C. (2022). B cell contribution to immunometabolic dysfunction and impaired immune responses in obesity. Clin. Exp. Immunol..

[41] O’Shea D., Hogan A.E. (2019). Dysregulation of natural killer cells in obesity. Cancers.

[42] Porsche C.E., Delproposto J.B., Patrick E., Zamarron B.F., Lumeng C.N. (2020). Adipose tissue dendritic cell signals are required to maintain T cell homeostasis and obesity-induced expansion. Mol. Cell Endocrinol..

[43] Cottam M.A., Caslin H.L., Winn N.C., Hasty A.H. (2022). Multiomics reveals persistence of obesity-associated immune cell phenotypes in adipose tissue during weight loss and weight regain in mice. Nat. Commun..

[44] Hildreth A.D., Ma F., Wong Y.Y., Sun R., Pellegrini M., O’Sullivan T.E. (2021). Single-cell sequencing of human white adipose tissue identifies new cell states in health and obesity. Nat. Immunol..

[45] Massier L., Jalkanen J., Elmastas M., Zhong J., Wang T., Nono Nankam P.A., Frendo-Cumbo S., Bäckdahl J., Subramanian N., Sekine T. (2023). An integrated single cell and spatial transcriptomic map of human white adipose tissue. Nat. Commun..

[46] Weinstock A., Brown E.J., Garabedian M.L., Pena S., Sharma M., Lafaille J., Moore K.J., Fisher E.A. (2019). Single-Cell RNA Sequencing of Visceral Adipose Tissue Leukocytes Reveals that Caloric Restriction Following Obesity Promotes the Accumulation of a Distinct Macrophage Population with Features of Phagocytic Cells. Immunometabolism.

[47] Maniyadath B., Zhang Q., Gupta R.K., Mandrup S. (2023). Adipose tissue at single-cell resolution. Cell Metab..

[48] Yi T., Wu S., Yang Y., Li X., Yang S., Zhang Y., Zhang L., Hu Y., Zhang G., Li J. (2025). Single-nucleus RNA sequencing reveals dynamic changes in the microenvironment of visceral adipose tissue and metabolic characteristics after cold exposure. Front. Endocrinol..

[49] So J., Strobel O., Wann J., Kim K., Paul A., Acri D.J., Dabin L.C., Kim J., Peng G., Roh H.C. (2025). Robust single-nucleus RNA sequencing reveals depot-specific cell population dynamics in adipose tissue remodeling during obesity. Elife.

[50] Baker R.G., Hayden M.S., Ghosh S. (2011). NF-κB, inflammation, and metabolic disease. Cell Metab..

[51] Hirosumi J., Tuncman G., Chang L., Görgün C.Z., Uysal K.T., Maeda K., Karin M., Hotamisligil G.S. (2002). A central role for JNK in obesity and insulin resistance. Nature.

[52] Cai D., Yuan M., Frantz D.F., Melendez P.A., Hansen L., Lee J., Shoelson S.E. (2005). Local and systemic insulin resistance resulting from hepatic activation of IKK-beta and NF-kappaB. Nat. Med..

[53] Cawthorn W.P., Sethi J.K. (2008). TNF-alpha and adipocyte biology. FEBS Lett..

[54] Nov O., Kohl A., Lewis E.C., Bashan N., Dvir I., Ben-Shlomo S., Fishman S., Wueest S., Konrad D., Rudich A. (2010). Interleukin-1beta may mediate insulin resistance in liver-derived cells in response to adipocyte inflammation. Endocrinology.

[55] Stanford K.I., Middelbeek R.J., Townsend K.L., Lee M.Y., Takahashi H., So K., Hitchcox K.M., Markan K.R., Hellbach K., Hirshman M.F. (2015). A novel role for subcutaneous adipose tissue in exercise-induced improvements in glucose homeostasis. Diabetes.

[56] Mauer J., Chaurasia B., Goldau J., Vogt M.C., Ruud J., Nguyen K.D., Theurich S., Hausen A.C., Schmitz J., Brönneke H.S. (2014). Signaling by IL-6 promotes alternative activation of macrophages to limit endotoxemia and obesity-associated resistance to insulin. Nat. Immunol..

[57] Molofsky A.B., Nussbaum J.C., Liang H.E., Van Dyken S.J., Cheng L.E., Mohapatra A., Chawla A., Locksley R.M. (2013). Innate lymphoid type 2 cells sustain visceral adipose tissue eosinophils and alternatively activated macrophages. J. Exp. Med..

[58] Feuerer M., Herrero L., Cipolletta D., Naaz A., Wong J., Nayer A., Lee J., Goldfine A.B., Benoist C., Shoelson S. (2009). Lean, but not obese, fat is enriched for a unique population of regulatory T cells that affect metabolic parameters. Nat. Med..

[59] Nishimura S., Manabe I., Nagasaki M., Eto K., Yamashita H., Ohsugi M., Otsu M., Hara K., Ueki K., Sugiura S. (2009). CD8+ effector T cells contribute to macrophage recruitment and adipose tissue inflammation in obesity. Nat. Med..

[60] Talukdar S., Oh D.Y., Bandyopadhyay G., Li D., Xu J., McNelis J., Lu M., Li P., Yan Q., Zhu Y. (2012). Neutrophils mediate insulin resistance in mice fed a high-fat diet through secreted elastase. Nat. Med..

[61] Cinti S., Mitchell G., Barbatelli G., Murano I., Ceresi E., Faloia E., Wang S., Fortier M., Greenberg A.S., Obin M.S. (2005). Adipocyte death defines macrophage localization and function in adipose tissue of obese mice and humans. J. Lipid Res..

[62] Winer S., Chan Y., Paltser G., Truong D., Tsui H., Bahrami J., Dorfman R., Wang Y., Zielenski J., Mastronardi F. (2009). Normalization of obesity-associated insulin resistance through immunotherapy. Nat. Med..

[63] Wu D., Molofsky A.B., Liang H.-E., Ricardo-Gonzalez R.R., Jouihan H.A., Bando J.K., Chawla A., Locksley R.M. (2011). Eosinophils Sustain Adipose Alternatively Activated Macrophages Associated with Glucose Homeostasis. Science.

[64] Jaitin D.A., Adlung L., Thaiss C.A., Weiner A., Li B., Descamps H., Lundgren P., Bleriot C., Liu Z., Deczkowska A. (2019). Lipid-Associated Macrophages Control Metabolic Homeostasis in a Trem2-Dependent Manner. Cell.

[65] Hill D.A., Lim H.W., Kim Y.H., Ho W.Y., Foong Y.H., Nelson V.L., Nguyen H.C.B., Chegireddy K., Kim J., Habertheuer A. (2018). Distinct macrophage populations direct inflammatory versus physiological changes in adipose tissue. Proc. Natl. Acad. Sci. USA.

[66] Thaler J.P., Yi C.X., Schur E.A., Guyenet S.J., Hwang B.H., Dietrich M.O., Zhao X., Sarruf D.A., Izgur V., Maravilla K.R. (2012). Obesity is associated with hypothalamic injury in rodents and humans. J. Clin. Investig..

[67] Valdearcos M., Xu A.W., Koliwad S.K. (2015). Hypothalamic inflammation in the control of metabolic function. Annu. Rev. Physiol..

[68] Gao Y., Ottaway N., Schriever S.C., Legutko B., García-Cáceres C., de la Fuente E., Mergen C., Bour S., Thaler J.P., Seeley R.J. (2014). Hormones and diet, but not body weight, control hypothalamic microglial activity. Glia.

[69] Jais A., Brüning J.C. (2017). Hypothalamic inflammation in obesity and metabolic disease. J. Clin. Investig..

[70] Maslennikov R., Efremova I., Ivashkin V., Zharkova M., Poluektova E., Shirokova E., Ivashkin K. (2022). Effect of probiotics on hemodynamic changes and complications associated with cirrhosis: A pilot randomized controlled trial. World J. Hepatol..

[71] Galaris A., Fanidis D., Stylianaki E.-A., Harokopos V., Kalantzi A.-S., Moulos P., Dimas A.S., Hatzis P., Aidinis V. (2022). Obesity Reshapes the Microbial Population Structure Along the Gut-Liver-Lung Axis in Mice. Biomedicines.

[72] Santos-Paulo S., Costello S.P., Forster S.C., Travis S., Bryant R.V. (2021). The Gut Microbiota as a Therapeutic Target for Obesity: A Scoping Review. Nutr. Res. Rev..

[73] Hossain F., Majumder S., David J., Bunnell B.A., Miele L. (2021). Obesity Modulates the Gut Microbiome in Triple-Negative Breast Cancer. Nutrients.

[74] Bawaneh A., Wilson A.S., Levi N., Howard-McNatt M., Chiba A., Soto-Pantoja D.R., Cook K.L. (2022). Intestinal Microbiota Influence Doxorubicin Responsiveness in Triple-Negative Breast Cancer. Cancers.

[75] Longo S., Rizza S., Federici M. (2023). Microbiota-Gut-Brain Axis: Relationships Among the Vagus Nerve, Gut Microbiota, Obesity, and Diabetes. Acta Diabetol..

[76] Boicean A., Ichim C., Sasu S.-M., Todor S.B. (2025). Key Insights into Gut Alterations in Metabolic Syndrome. J. Clin. Med..

[77] Zatterale F., Longo M., Naderi J., Raciti G.A., Desiderio A., Miele C., Beguinot F. (2019). Chronic Adipose Tissue Inflammation Linking Obesity to Insulin Resistance and Type 2 Diabetes. Front. Physiol..

[78] Savulescu-Fiedler I., Mihalcea R., Dragosloveanu S., Scheau C., Baz R.O., Caruntu A., Scheau A.-E., Caruntu C., Benea S.N. (2024). The Interplay between Obesity and Inflammation. Life.

[79] Tylutka A., Morawin B., Wawrzyniak-Gramacka E., Wacka E., Nowicka W., Hiczkiewicz J., Zembron-Lacny A. (2022). Immunosenescence in Aging-Related Vascular Dysfunction. Int. J. Mol. Sci..

[80] Chen Y., Zhu C., Chen Y., Wang N., Qin L., Han B., Zhao L., Chen C., Zhai H., Lu Y. (2018). The Association of Thyroid Nodules with Metabolic Status: A Cross-Sectional SPECT-China Study. Int. J. Endocrinol..

[81] Moffa S., Mezza T., Cefalo C., Cinti F., Impronta F., Sorice G.P., Santoro A., Giuseppe G.D., Pontecorvi A., Giaccari A. (2019). The Interplay Between Immune System and Microbiota in Diabetes. Mediat. Inflamm..

[82] Bashir A., Duseja A., De A., Mehta M., Tiwari P. (2022). Non-alcoholic fatty liver disease development: A multifactorial pathogenic phenomena. Liver Res..

[83] Sutti S., Albano E. (2020). Adaptive immunity: An emerging player in the progression of NAFLD. Nat. Rev. Gastroenterol. Hepatol..

[84] Berbudi A., Khairani S., Tjahjadi A.I. (2025). Interplay Between Insulin Resistance and Immune Dysregulation in Type 2 Diabetes Mellitus: Implications for Therapeutic Interventions. Immuno Targets Ther..

[85] Ma J., Li Y., Yang X., Liu K., Zhang X., Zuo X., Ye R., Wang Z., Shi R., Meng Q. (2023). Signaling pathways in vascular function and hypertension: Molecular mechanisms and therapeutic interventions. Signal Transduct. Target. Ther..

[86] Muralitharan R.R., Marques F.Z., O’Donnell J.A. (2024). Recent advancements in targeting the immune system to treat hypertension. Eur. J. Pharmacol..

[87] Li K., Shi H., Zhang B., Ou X., Ma Q., Chen Y., Shu P., Li D., Wang Y. (2021). Myeloid-derived suppressor cells as immunosuppressive regulators and therapeutic targets in cancer. Signal Transduct. Target. Ther..

[88] Chen W., Pang Y. (2021). Metabolic Syndrome and PCOS: Pathogenesis and the Role of Metabolites. Metabolites.

[89] Wang J., Yin T., Liu S. (2023). Dysregulation of immune response in PCOS organ system. Front. Immunol..

[90] Zhu M., Xu Y., Li C., Lu Z., Bi K., Wang K., Guo P., Jiang H., Cao Y. (2021). Involvement of impaired CD8(+) mucosal-associated invariant T cells and myeloid-derived suppressor cells in polycystic ovary syndrome. Reprod. Biol. Endocrinol..

[91] Hall J.E., Mouton A.J., da Silva A.A., Omoto A.C.M., Wang Z., Li X., do Carmo J.M. (2021). Obesity, kidney dysfunction, and inflammation: Interactions in hypertension. Cardiovasc. Res..

[92] Zoccali C., Mallamaci F., Adamczak M., de Oliveira R.B., Massy Z.A., Sarafidis P., Agarwal R., Mark P.B., Kotanko P., Ferro C.J. (2023). Cardiovascular complications in chronic kidney disease: A review from the European Renal and Cardiovascular Medicine Working Group of the European Renal Association. Cardiovasc. Res..

[93] Gabryelska A., Łukasik Z.M., Makowska J.S., Białasiewicz P. (2018). Obstructive Sleep Apnea: From Intermittent Hypoxia to Cardiovascular Complications via Blood Platelets. Front. Neurol..

[94] Lee S.A., Riella L.V. (2024). Narrative Review of Immunomodulatory and Anti-inflammatory Effects of Sodium-Glucose Cotransporter 2 Inhibitors: Unveiling Novel Therapeutic Frontiers. Kidney Int. Rep..

[95] Androutsakos T., Nasiri-Ansari N., Bakasis A.D., Kyrou I., Efstathopoulos E., Randeva H.S., Kassi E. (2022). SGLT-2 Inhibitors in NAFLD: Expanding Their Role beyond Diabetes and Cardioprotection. Int. J. Mol. Sci..

[96] Ridker P.M., Everett B.M., Thuren T., MacFadyen J.G., Chang W.H., Ballantyne C., Fonseca F., Nicolau J., Koenig W., Anker S.D. (2017). Antiinflammatory Therapy with Canakinumab for Atherosclerotic Disease. N. Engl. J. Med..

[97] Zhang Z., Zhao L., Zhou X., Meng X., Zhou X. (2022). Role of inflammation, immunity, and oxidative stress in hypertension: New insights and potential therapeutic targets. Front. Immunol..

[98] Henein M.Y., Vancheri S., Longo G., Vancheri F. (2022). The Role of Inflammation in Cardiovascular Disease. Int. J. Mol. Sci..

[99] Singh S., Pal N., Shubham S., Sarma D.K., Verma V., Marotta F., Kumar M. (2023). Polycystic Ovary Syndrome: Etiology, Current Management, and Future Therapeutics. J. Clin. Med..

[100] Maringhini S., Zoccali C. (2024). Chronic Kidney Disease Progression-A Challenge. Biomedicines.

[101] Ryan S., Taylor C.T., McNicholas W.T. (2005). Selective activation of inflammatory pathways by intermittent hypoxia in obstructive sleep apnea syndrome. Circulation.

[102] Varra F.-N., Varras M., Varra V.-K., Theodosis-Nobelos P. (2024). Molecular and pathophysiological relationship between obesity and chronic inflammation in the manifestation of metabolic dysfunctions and their inflammation-mediating treatment options. Mol. Med. Rep..

[103] Vick L.V., Canter R.J., Monjazeb A.M., Murphy W.J. Multifaceted effects of obesity on cancer immunotherapies: Bridging preclinical models and clinical data. Proceedings of the Seminars in Cancer Biology.

[104] Garg S.S., Kushwaha K., Dubey R., Gupta J. (2023). Association between obesity, inflammation and insulin resistance: Insights into signaling pathways and therapeutic interventions. Diabetes Res. Clin. Pract..

[105] Petrelli F., Cortellini A., Indini A., Tomasello G., Ghidini M., Nigro O., Salati M., Dottorini L., Iaculli A., Varricchio A. (2020). Obesity paradox in patients with cancer: A systematic review and meta-analysis of 6,320,365 patients. MedRxiv.

[106] Yan J., Wang Y., Mu Z., Han X., Bi L., Wang X., Song P., Kang Y., Wang L., Zhang X. (2023). Gold Nanobipyramid-Mediated Apoptotic Camouflage of Adipocytes for Obesity Immunotherapy. Adv. Mater..

[107] Hemachandra S., Rathnayake S.N., Jayamaha A.A., Francis B.S., Welmillage D., Kaur D.N., Zaw H.K., Zaw L.T., Chandra H.A., Abeysekera M.E. (2025). Fecal Microbiota Transplantation as an Alternative Method in the Treatment of Obesity. Cureus.

[108] Khan S., Luck H., Winer S., Winer D.A. (2021). Emerging concepts in intestinal immune control of obesity-related metabolic disease. Nat. Commun..

[109] Du W., Zou Z.P., Ye B.C., Zhou Y. (2025). Gut microbiota and associated metabolites: Key players in high-fat diet-induced chronic diseases. Gut Microbes.

[110] Lillich F.F., Imig J.D., Proschak E. (2021). Multi-target approaches in metabolic syndrome. Front. Pharmacol..

[111] Wang Z., Yang B. (2022). Polypharmacology in clinical applications: Metabolic Disease Polypharmacology. Polypharmacology: Principles and Methodologies.

[112] Czókolyová M., Pusztai A., Végh E., Horváth Á., Szentpéteri A., Hamar A., Szamosi S., Hodosi K., Domján A., Szántó S. (2021). Changes of metabolic biomarker levels upon one-year anti-TNF-α therapy in rheumatoid arthritis and ankylosing spondylitis: Associations with vascular pathophysiology. Biomolecules.

[113] Osborn O., Brownell S., Sanchez-Alavez M., Salomon D., Gram H., Bartfai T. (2008). Treatment with an Interleukin 1 beta antibody improves glycemic control in diet-induced obesity. Cytokine.

[114] Park C.-S., Shastri N. (2022). The role of T cells in obesity-associated inflammation and metabolic disease. Immune Netw..

[115] Aguilar E.G., Murphy W.J. (2018). Obesity induced T cell dysfunction and implications for cancer immunotherapy. Curr. Opin. Immunol..

[116] Nguyen K.D., Qiu Y., Cui X., Goh Y.P., Mwangi J., David T., Mukundan L., Brombacher F., Locksley R.M., Chawla A. (2011). Alternatively activated macrophages produce catecholamines to sustain adaptive thermogenesis. Nature.

[117] Woodall M.J., Neumann S., Campbell K., Pattison S.T., Young S.L. (2020). The effects of obesity on anti-cancer immunity and cancer immunotherapy. Cancers.

[118] Vallianou N., Stratigou T., Christodoulatos G.S., Dalamaga M. (2019). Understanding the role of the gut microbiome and microbial metabolites in obesity and obesity-associated metabolic disorders: Current evidence and perspectives. Curr. Obes. Rep..

[119] Geng J., Ni Q., Sun W., Li L., Feng X. (2022). The links between gut microbiota and obesity and obesity related diseases. Biomed. Pharmacother..

[120] Farzaei M.H., Singh A.K., Kumar R., Croley C.R., Pandey A.K., Coy-Barrera E., Kumar Patra J., Das G., Kerry R.G., Annunziata G. (2019). Targeting inflammation by flavonoids: Novel therapeutic strategy for metabolic disorders. Int. J. Mol. Sci..

[121] Clemmensen C., Finan B., Müller T.D., DiMarchi R.D., Tschöp M.H., Hofmann S.M. (2019). Emerging hormonal-based combination pharmacotherapies for the treatment of metabolic diseases. Nat. Rev. Endocrinol..

[122] De Frel D.L., Atsma D.E., Pijl H., Seidell J.C., Leenen P.J., Dik W.A., Van Rossum E.F. (2020). The impact of obesity and lifestyle on the immune system and susceptibility to infections such as COVID-19. Front. Nutr..

[123] Qasim A., Turcotte M., de Souza R.J., Samaan M.C., Champredon D., Dushoff J., Speakman J.R., Meyre D. (2018). On the origin of obesity: Identifying the biological, environmental and cultural drivers of genetic risk among human populations. Obes. Rev..

[124] Apolzan J.W., Venditti E.M., Edelstein S.L., Knowler W.C., Dabelea D., Boyko E.J., Pi-Sunyer X., Kalyani R.R., Franks P.W., Srikanthan P. (2019). Long-term weight loss with metformin or lifestyle intervention in the diabetes prevention program outcomes study. Ann. Intern. Med..

[125] Wang J.-Y., Wang Q.-W., Yang X.-Y., Yang W., Li D.-R., Jin J.-Y., Zhang H.-C., Zhang X.-F. (2023). GLP-1 receptor agonists for the treatment of obesity: Role as a promising approach. Front. Endocrinol..

[126] Khoo B., Tan T.M.-M. (2020). Combination gut hormones: Prospects and questions for the future of obesity and diabetes therapy. J. Endocrinol..

[127] Halabitska I., Babinets L., Oksenych V., Kamyshnyi O. (2024). Diabetes and osteoarthritis: Exploring the interactions and therapeutic implications of insulin, metformin, and GLP-1-based interventions. Biomedicines.

[128] Pandita S. (2023). Ambiguous Effects of Obesity on Cancer Prognosis and Treatment Response: A Narrative Review. EMJ.

[129] Boland L., Bitterlich L.M., Hogan A.E., Ankrum J.A., English K. (2022). Translating MSC therapy in the age of obesity. Front. Immunol..

[130] Li X., Lau S.K., Woo P.C. (2020). Fungal infection risks associated with the use of cytokine antagonists and immune checkpoint inhibitors. Exp. Biol. Med..

[131] Bakos O., Lawson C., Rouleau S., Tai L.-H. (2018). Combining surgery and immunotherapy: Turning an immunosuppressive effect into a therapeutic opportunity. J. Immunother. Cancer.

[132] Fernández-Ruiz M., Aguado J.M. (2018). Risk of infection associated with anti-TNF-α therapy. Expert Rev. Anti-Infect. Ther..

[133] Peiró C., Lorenzo Ó., Carraro R., Sánchez-Ferrer C.F. (2017). IL-1β inhibition in cardiovascular complications associated to diabetes mellitus. Front. Pharmacol..

[134] Amjad S., Liaqat I., Amjad Z., Fatima S., Hashmi M., Khan W., Nasir M. (2024). Genomic Insights: Predicting Obesity through AI and Machine Learning. Complementary and Alternative Medicine: Non–Conventional.

[135] Peng J., Jury E.C., Dönnes P., Ciurtin C. (2021). Machine learning techniques for personalised medicine approaches in immune-mediated chronic inflammatory diseases: Applications and challenges. Front. Pharmacol..

[136] Popa M.L., Ichim C., Anderco P., Todor S.B., Pop-Lodromanean D. (2025). MicroRNAs in the Diagnosis of Digestive Diseases: A Comprehensive Review. J. Clin. Med..

